# The Vγ9Vδ2 T Cell Antigen Receptor and Butyrophilin-3 A1: Models of Interaction, the Possibility of Co-Evolution, and the Case of Dendritic Epidermal T Cells

**DOI:** 10.3389/fimmu.2014.00648

**Published:** 2014-12-19

**Authors:** Mohindar M. Karunakaran, Thomas Herrmann

**Affiliations:** ^1^Department of Medicine, Institute for Virology and Immunobiology, University of Würzburg, Würzburg, Germany

**Keywords:** γδ T cell, Vγ9Vδ2 T cell, phosphoantigen, BTN3, alpaca, co-evolution, DETC and Skint1

## Abstract

Most circulating human gamma delta T cells are Vγ9Vδ2 T cells. Their hallmark is the expression of T cell antigen receptors (TCR) whose γ-chains show a Vγ9-JP (Vγ2-Jγ1.2) rearrangement and are paired with Vδ2-containing δ-chains, a dominant TCR configuration, which until recently seemed to occur in primates only. Vγ9Vδ2 T cells respond to phosphoantigens (PAg) such as (*E*)-4-Hydroxy-3-methyl-but-2-enyl pyrophosphate (HMBPP), which is produced by many pathogens and isopentenyl pyrophosphate (IPP), which accumulates in certain tumors or cells treated with aminobisphosphonates such as zoledronate. A prerequisite for PAg-induced activation is the contact of Vγ9Vδ2 T cells with cells expressing butyrophilin-3 A1 (BTN3A1). We will first critically review models of how BTN3 might act in PAg-mediated Vγ9Vδ2 T cell activation and then address putative co-evolution of Vγ9, Vδ2, and *BTN3* genes. In those rodent and lagomorphs used as animal models, all three genes are lost but a data-base analysis showed that they emerged together with placental mammals. A strong concomitant conservation of functional Vγ9, Vδ2, and *BTN3* genes in other species suggests co-evolution of these three genes. A detailed analysis was performed for the new world camelid alpaca (*Vicugna pacos*). It provides an excellent candidate for a non-primate species with presumably functional Vγ9Vδ2 T cells since TCR rearrangements share features characteristic for PAg-reactive primate Vγ9Vδ2 TCR and proposed PAg-binding sites of BTN3A1 have been conserved. Finally, we analyze the possible functional relationship between the butyrophilin-family member Skint1 and the γδ TCR-V genes used by murine dendritic epithelial T cells (DETC). Among placental mammals, we identify five rodents, the cow, a bat, and the cape golden mole as the only species concomitantly possessing potentially functional homologs of murine Vγ3, Vδ4 genes, and *Skint1* gene and suggest to search for DETC like cells in these species.

## αβ T Cells and γδ T Cells

Jawed vertebrates (Gnathostomata) possess lymphocytes expressing Ig-domain containing antigen-receptors, whose highly diverse antigen-binding sites are generated by RAG-dependent somatic recombination of genes encoding for an antigen-binding variable-domain. Such receptors are broadly classified into three types. αβ and γδ T cell antigen receptors (TCR), which are encoded by αβ and γδ genes, respectively, and the B cell antigen receptors, which are encoded by immunoglobulin heavy and light chain genes (1–3). The exact sequence of emergence of the antigen-receptor genes is controversial. One view is that the γδ TCR encoding genes originally constituted a receptor for soluble antigens, which is primordial to the MHC-restricted αβ TCR, BCR, and antibodies (2, 4). Alternatively, αβ and γδ TCR-genes may have emerged from a common ancestor (5). An interesting case of convergent evolution has occurred in jawless vertebrates (Agnatha). Their lymphocytes express antigen-receptors completely distinct from those of Gnathostomata both in terms of molecular composition (leucine rich repeats instead of Ig domains) and genetic basis of diversity generation [Cytidine deaminase (AID) dependent gene conversion]. Three distinct lineages of lymphocytes expressing distinct antigen receptors variable leukocyte receptors (VLR) (6, 7) have been identified: T-like lymphocytes maturing in the thymus express VLRA and VLRC while B-like lymphocytes produce VLRB as soluble antigen-receptors (8).

Among T cells, those which confer adaptive immunity are MHC-restricted T cells. They express αβ TCR, which bind to complexes of polymorphic MHC molecules and peptide antigens. Their diversity is generated by RAG-mediated recombination of V(D)J genes of both chains. The diversity of their third complementarity-determining regions (CDR3) which are encoded by the VαJα and VβDβJβ transition is further increased by joining flexibility and insertion of P and N-nucleotides. The final composition of TCR specificities (T cell repertoire) is shaped by intrathymic positive and negative selection guided by anatomically controlled presentation of peptide-MHC complexes and avidity of binding of the emerging TCR to those complexes (9). A highly conserved (2, 3), although not absolute feature in all vertebrate species (10), is the division of mature T cells as in MHC-class I restricted CD8 T cells, which exert killer function and MHC-class II restricted CD4 T cells, which regulate immune functions (2, 3). In spite of a likely co-evolution of the peptide presenting MHC molecules with Vα and Vβ TCR genes, it is not possible to predict MHC-class restriction or antigen-specificity of a given T cell from usage of certain TCR genes (11–13).

Alongside “conventional” MHC-restricted αβ T cells, several T cell populations exist with specificities for ligands other than MHC-peptide complexes. These “non-conventional” T cells can express αβ or γδ TCR. The generation of their TCR diversity follows the same genetic mechanisms as for MHC-restricted αβ TCR but in contrast to MHC-restricted T cells TCR-gene usage can be predictive for ontogeny, homing, and effector functions and is used to define distinct T cell subpopulations (12, 14–17). The best understood population of non-conventional T cells are the CD1d-restricted invariant NKT cells (17, 18). Their TCRα chains are invariant with a characteristic VαJα rearrangement and pair with β-chains of restricted Vβ gene usage. The iNKT TCRs bind in a highly conserved manner to complexes of the non-polymorphic MHC-class I like CD1d molecule and microbial or host cell glycolipids. Thus, functionally they resemble pattern recognition receptors of innate immune cells, which do not discriminate between highly variable antigens but recognize molecular patterns (17, 18). In contrast to MHC-restricted αβ T cells, which at least in mammals differentiate into CD8 killer T cells and CD4 helper and regulatory T cells, subpopulations of non-conventional T cells vary strongly between phylogenetic groups, e.g., many mammals lack iNKT cells as well as the restricting CD1d molecule (19, 20) but even closely related species such as mouse and rat differ dramatically in terms of iNKT-cell frequency although the respective genes for iNKT TCR and CD1d are highly conserved (21).

γδ T cells differ strongly between taxa. This difference can be rather global as in case of γδ T cells whose frequencies among blood T cells vary between 1 and 5% in man and mouse to 50% in ruminants (22). The presence or absence of entire populations defined by their TCR gene usage is even more striking (14, 23–25). Two such cases are addressed in this review: at first the human Vγ9Vδ2 T cells, which have so far only been found in higher primates (26). For their function, associated with various microbial and host metabolites, the molecule butyrophilin-3A1 is mandatory and this article will address the putative function of BTN3A1 and current evidence for a co-evolution of Vγ9Vδ2 TCR and BTN3A1. Secondly, we provide a first analysis of phylogeny of Vγ and Vδ genes constituting the TCR of murine dendritic epidermal T cell receptor (DETC) (27–30) and *Skint1*, a member of the butyrophilin family, which is critical during the ontogeny and function of these cells (31).

## Vγ9Vδ2 T Cells: TCR and Phosphoantigen Reactivity

The vast majority of human blood γδ T cells respond to so called “Phosphoantigens” (PAg) (32–34). Their TCR share a characteristic Vγ9JP (alternatively designated as Vγ2Jγ1.2) rearrangement (35–38) and Vδ2-containing chains (35) bearing one of the hydrophobic amino acids (38, 39): Leucine (L), Isoleucine (I), Valine (V), or Glycine (G) at position 97. Unless explicitly mentioned PAg-reactive T cells and Vγ9Vδ2 T cells will be used as synonyms in this article.

Freshly isolated Vγ9Vδ2 T cells share functional features with TH1 cells, CD8 T cells, and NK cells (40) but upon culture they can differentiate to TH17 like (41), and to professional antigen-presenting cells (42). Apart from killing or cytokine release, they promote and regulate immune responses by crosstalk with dendritic cells (43), NK cells (44), and monocytes (45). Numerous preclinical and clinical studies demonstrated their therapeutic potential for treatment of tumors (46, 47) and infection (48, 49). The antigen-dependent activation of Vγ9Vδ2 T cells is strongly modulated by additional receptors (50) including inhibitory and activating NK-cell receptors (51, 52). In case of NKG2D even a direct triggering of some effector functions is possible (53).

PAg are products of isoprenoid synthesis, which specifically activate Vγ9Vδ2 T cells. The building blocks of isoprenoid synthesis are isopentenyl pyrophosphate (IPP) and its isomer dimethylallyl pyrophosphate. Both are weak PAg (54, 55) and 1000- to 10000-fold less potent than the strongest naturally occurring PAg (*E*)-4-Hydroxy-3-methyl-but-2-enyl pyrophosphate (HMBPP) (26, 55, 56). HMBPP is the immediate precursor in the synthesis of IPP by the non-mevalonate pathway also known as DOXP, MEP, or Rohmer pathway. The non-mevalonate-pathway is restricted to eubacteria, cyanobacteria, plants, and apicomplexan protozoa (26, 56), which may have adopted this pathway by endosymbiosis of precursors of chloroplasts and apicoplast, respectively (57, 58). HMBPP is the driving force of a massive Vγ9Vδ2 T cell expansion in infections with HMBPP producing parasites or bacteria, which can lead to an increase of Vγ9Vδ2 T cells from 1 to 5% of blood T cells to more than 50% (26, 49). In mammals and most other animals, IPP is synthesized via the mevalonate pathway whose manipulation can render human cells into Vγ9Vδ2 T cell activators. Cells pulsed with aminobisphosphonates (e.g., zoledronate or pamidronate) become potent activators of primary Vγ9Vδ2 T cells (59, 60) and of Vγ9Vδ2 TCR transductants (61) most likely in consequence of IPP accumulation after inhibition of the IPP metabolizing farnesyl diphosphate synthase (FPPS) (59, 62). The same effects are seen by inhibiting expression of FPPS (63, 64) and of isopentenyl diphosphate isomerase (64). Other modes of activation by IPP accumulation are activation (65) or over-expression of HMG-CoA reductase (66), which is the rate-controlling enzyme upstream of IPP synthesis, and finally inhibition of FPPS by alkylamines (67, 68).

Altogether the Vγ9Vδ2 TCR acts as a kind of pattern recognition receptor, which senses microbial infections as well as metabolic changes found in transformed, infected, or drug treated host cells (69). This reactivity can be harnessed clinically since remission of certain tumor entities after Vγ9Vδ2 T cell activation was observed in clinical trials (46, 47, 59, 60) and it may even contribute to the beneficial effects of zoledronate seen upon treatment of premenopausal women with early-stage breast cancer (70).

Some tumors such as the human B cell lymphoma Daudi (71, 72) spontaneously activate Vγ9Vδ2 T cells. This activation depends on intracellular accumulation of IPP and can be abolished by statins, which inhibit the HMG-CoA reductase and consequently also IPP synthesis (66). In contrast, still unclear despite intensive investigation is the contribution and the mechanistic basis of Vγ9Vδ2 T cell activation by the pro-apoptotic IPP metabolite ApppI (triphosphoric acid 1-adenosin-50-yl ester 3-(3-methylbut-3-enyl) ester), which is synthesized from IPP and AMP by aminoacyl-tRNA-synthetases (73, 74). Also not clear is how PAg-action is associated with the reported binding of the Vγ9Vδ2 TCR G115 to ectopically expressed F1-ATPase (74, 75).

## BTN3 is Mandatory for PAg-Mediated Activation of Vγ9Vδ2 T Cells

PAg act not as soluble molecules but need to be “presented” by cells of human or primate origin (76). This can be seen as evidence for species-specific molecules, which could be PAg-presenting molecules and/or for molecules with special co-stimulatory or cell–cell interaction mediating properties (77, 78). Attempts to identify PAg-binding cell surface molecules by biochemical means, e.g., with the help of photo-activated PAg analogs have failed so far (79, 80) although direct binding of tetramers of rhesus monkey Vγ9Vδ2 TCR to HMBPP pulsed primate dendritic cells was reported (81).

The major breakthrough in defining species-specific molecular requirements for Vγ9Vδ2 T cell activation by PAg was the identification of the pivotal role of Butyrophilin 3 (BTN3) in modulating PAg induced responses (82). BTN3 – also named CD277 – is a membrane protein, which belongs to the butyrophilin family and shares similarity with the B7 family (83). In human beings, three isoforms of BTN3 named BTN3A1, BTN3A2, and BTN3A3 exist whose genes localize to the telomeric end of the MHC complex on the short arm of human chromosome 6 (84). The extracellular domain of BTN3 (BTN3-ED) consists of an N-terminal IgV-like domain (V-domain) and a membrane proximal IgC-like domain (C-domain). The BTN3-ED of the three isoforms is extremely similar and cannot be discriminated by the available antibodies (82, 83). The intracellular domain (ID) of BTN3A1 and BTN3A3 belongs to the B30.2 family (85, 86) and is often found in proteins involved in innate immunity. This includes even molecules encoded by genes found in the hypothetical “proto MHC” of placozoa, which form the most basal branch of Metazoa (87). The BTN3A2 molecule has a truncated intracellular B30.2-negative domain. So far the binding of BTN3 to Vγ9Vδ2 TCR is controversial and a natural ligand or counter-receptor of the BTN3-extracellular domain has not been found yet. Moreover, CD277 antibodies modulate not only responses of Vγ9Vδ2 T cells but also those of αβ T cells and NK cells (88, 89).

The first evidence for a critical role of BTN3 in Vγ9Vδ2 T cell activation came from the agonistic action of the CD277-specific monoclonal antibody (mAb) 20.1 on Vγ9Vδ2 T cells and other CD277-specific antibodies, such as mAb 103.2, being antagonists for PAg-mediated activation (82). Co-cultures of Vγ9Vδ2 T cells or human Vγ9Vδ2 TCR transductants with mAb-pulsed BTN3 positive cells suggested that mAbs work by binding to stimulatory or target cells and not to the human γδ T cells. This was formally proven by demonstrating that mAb 20.1 activates BTN3-negative murine Vγ9Vδ2 TCR transductants in co-cultures with human Raji cells (82). These reporter cells were either mouse hybridoma BW58 (or C58 α-β-) or the rat/mouse T cell hybridoma 53/4-transduced with a Vγ9Vδ2 TCR (TCR-MOP) (82, 90, 91). Interestingly, the PAg-induced IL-2 production by both cell types depends strictly on provision of strong co-stimulatory signal. This can be achieved via ligation of a chimeric rat/mouse CD28 overexpressed on the surface of the reporter cell by CD80 or CD86 on the stimulatory human cell, which can be of natural origin or introduced by gene-transduction (91).

Activation by mAb 20.1 of TCR-MOP transductants but also of primary responder cells is usually less efficient than PAg-induced activation. Nevertheless, it has been shown for primary γδ T cells that PAg- and mAb 20.1-induced activation are quite similar with respect to TCR-mediated signals and phenotypic changes of the cells (92). Furthermore, activation by mAb 20.1 is resistant to statins and not accompanied by accumulation of IPP (80, 82).

## Models of PAg-Action: Allosteric Change vs. Antigen Presentation

At present, two major hypotheses to explain PAg-mediated activation of Vγ9Vδ2 T cells compete with each other. One suggests that PAg-binding to the ID of BTN3A1 provokes changes in the BTN3A1-ED either directly or with help of molecules recruited by the ID. These events are accompanied by a reduced mobility of cell surface BTN3A1 and are mandatory for binding of the Vγ9Vδ2 TCR to BTN3A1 or associated molecule(s) alone or in complex with BTN3. We will refer to this concept as the “allosteric model” (82, 93, 94). The other concept suggests a direct binding of BTN3A1-PAg complexes to the Vγ9Vδ2 TCR and describes BTN3A1 as an antigen-presenting molecule. This will be referred to as the “antigen-presentation model” (95).

### The allosteric model

The work of Harly et al. (82) describes not only the general importance of BTN3 for the PAg response but also that BTN3 isoforms differently support PAg-dependent Vγ9Vδ2 T cell activation while no such differences are found for mAb 20.1-induced activation. Evidence was obtained by comparing BTN3 isoform specific knockdown cells and transductants for their capacity to induce PAg-dependent stimulation of primary Vγ9Vδ2 T cells.

The same pattern was found for the reduction in BTN3-cell surface motility. In photobleaching experiments cells expressing BTN3A1 or BTN3A2 constructs were compared. Aminobisphophonate (pamidronate) pulsed BTN3A1 but not BTN3A2 transductants showed a clear mevastatin-sensitive decrease in cell surface mobility suggesting a PAg-induced BTN3 isoform related effect. In contrast, for mAb 20.1 no differences between the isoforms were revealed although it decreased motility even stronger than PAg. Further important insights came from structural and functional characterization of the BTN3-ED (93). In this study, soluble and crystallized BTN3-ED revealed no major difference between the three BTN3 isoforms and could consequently not help to explain their functional difference. More interestingly, these structural analyses revealed two types of BTN3-homodimers. One had a symmetric paralleled structure where both chains were fixed by interaction of their C-domains. The other one had an asymmetric head to tail conformation with contacts between V- and C-domain, respectively. Importantly, the agonistic but not the antagonistic antibody favors formation of the symmetric dimer. In addition, co-crystals with single chain (sc)20.1 revealed a conformational shift of the BTN3 dimer while sc103.2 has no such effect. These results together with other data lead to model where BTN3 and mAb 20.1 binds with a 2:1 stoichiometry and formed a BTN3 lattice at the cells surface. In contrast, intact mAb 103.2 binds with 1:1 stoichiometry and is expected to inhibit formation of such lattices while no inhibitory effects were seen with sc103.2.

**Figure 1:**
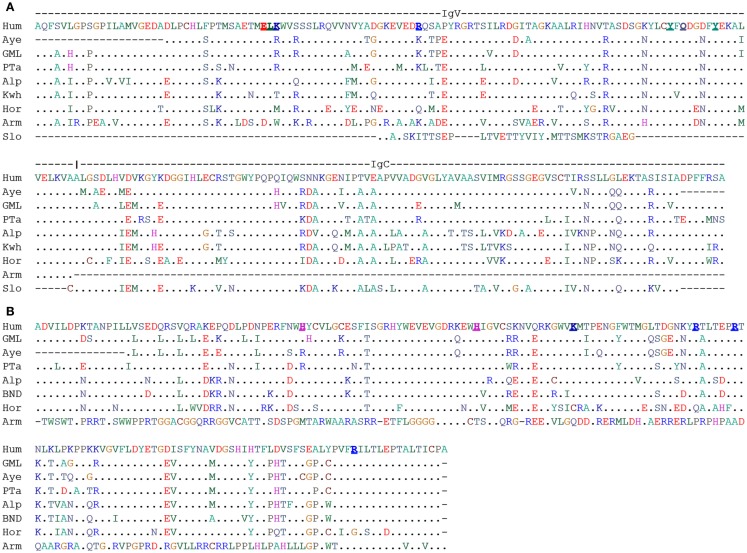
**Alignment of human BTN3A1 (V-C) and B30.2 domain**. **(A)** ClustalW2 amino acid alignment of V-C domains of human BTN3A1 extracellular domain with respective homologous sequence identified from WGS database at NCBI. Underlined bold amino acids of human BTN3A1-ED were predicted to interact with PAg (95). **(B)** ClustalW2 amino acid alignment of intracellular B30.2 domains of human BTN3A1 with respective homologous sequence. Underlined bold amino acids were of human BTN3A1-ID were predicted to interact with PAg (94). Species were abbreviated as Hum, Human; Aye, Aye-aye; GML, Gray mouse lemur; PTa, Philippine tarsier; Alp, Alpaca; Kwh, Killer whales; Hor, Horse; Arm, Armadillo; Slo, Sloth.

**Figure 2:**
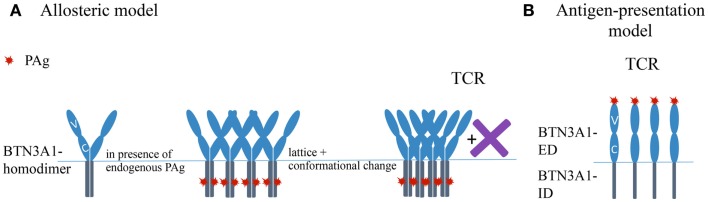
**Simplified sketches depicting the “allosteric model” and the “antigen-presenting model.”** Note that both models can be partially combined. For reasons of simplicity involvement of hypothetical additional molecule(s) (X) is only depicted were being absolutely necessary. **(A)** Sketch representing the allosteric model describing possible events involved in PAg mediated activation of Vγ9Vδ2 TCR. X – represents unidentified molecules, which might involve in mechanism. **(B)** Schematic representation of antigen-presentation model. TCR, T cell receptor; PAg, phosphoantigen.

Important clues how IDs might control PAg-dependent stimulation comes from recent crystallographic and functional studies (94). This study first identified by deletion and amino acid exchange mutants a region in the B30.2 domain as critical for PAg-action. Crystallographic analysis identified then a positively charged pocket in this region (the presumed contacts with the PAg are marked in Figure 1B), which could accommodate a PAg. Isothermal calorimetry demonstrated PAg-binding to recombinant BTN3A1-ID, which was considerably stronger for HMBPP than for IPP and was extinguished by the same mutants, which abolished Vγ9Vδ2 TCR activation by respective BTN3 transductants. The importance of the pocket is further corroborated by a single aa mutant in BTN3A1 (H351R), which destroyed the PAg-binding to BTN3A1-ID, the PAg-dependent activation of Vγ9Vδ2 T cells and the zoledronate-induced reduction of cell surface motility. Altogether, evidence is provided that changes induced by PAg-binding to BTN3A1-ID correlate with changes seen in BTN3A1 cell surface motility and PAg-induced Vγ9Vδ2 T cell activation (82, 93, 94). For illustrating the key points of this hypothesis a simplified version of the “allosteric model” is depicted in Figure 2A.

### The antigen-presenting model

Vavassori et al. used a different approach to identify the species-specific factor mandatory for PAg-mediated activation and describe BTN3A1 as an *antigen-presenting molecule* (95). Their approach takes advantage of the fact that mouse lacks a BTN3 ortholog. At first, murine reporter cells expressing a Vγ9Vδ2 TCR were used to screen mouse-human hybrid cell lines for their capacity to mediate PAg-dependent stimulation with the aim to map this trait to a part of the human genome. By analysis of several of such mouse-hybrid cell lines the telomeric 3–27 Mb region of the human chromosome 6p was found to be mandatory for PAg-presentation. This region comprises the entire MHC as well as the *BTN3A1* and *BTN3A2* but not the *BTN3A3* gene. Thus, genomic localization of the mandatory gene(s) is fully consistent with previously published data that BTN3A1 is mandatory for PAg-mediated activation. The genetic evidence for BTN3A1 as candidate for the molecule involved was further confirmed by knock down and over-expression experiments.

Interestingly, the reporter cells used in this study were not Vγ9Vδ2 TCR-transduced murine hybridoma cells as described above but Vγ9Vδ2 T lymphocytes generated from RAG knock-out mice transgenic for the Vγ9Vδ2 TCR B2G9, which were matured *in vivo* by administration of anti-CD3 mAb (95, 96). An important difference between data obtained with primary murine reporter cells expressing the Vγ9Vδ2 TCR B2G9 and Vγ9Vδ2 TCR-MOP transduced reporter cells is that the agonistic mAb 20.1 was not stimulatory but inhibitory for the transgenic mouse cells. First results of our group obtained with TCR transductants suggest that this difference reflects variation of the TCR clonotypes, which stands against the idea of mAb 20.1 being a general activator of Vγ9Vδ2 T cells. Nevertheless, to our knowledge, there is no published data on determination of frequencies of mAb 20.1 vs. PAg-reactive cells or direct comparison of sensitivity of different TCR clonotypes for either stimulus supporting this notion. If TCR clonotypes do indeed differ in their sensitivity to both types of stimuli, it would affect models on PAg or mAb 20.1 action. Our interpretation of the presumed clonal differences would rely on substrate competition and inherent qualities of different TCR clonotypes. In the former case, we hypothesize that upon treatment of cells with PAg or mAb 20.1 BTN3A1 adopts a new conformation, which somehow allows binding of Vγ9Vδ2 TCR to BTN3-ED-PAg or mAb complex or to BTN3-ED-associated cell surface molecules(s). This conformation could differ to some extent after exposure of the cell to PAg or mAb 20.1 whereby mAb 20.1 might inhibit conversion into the PAg induced conformation. As a result, some TCR clonotypes cannot bind to the mAb 20.1-induced conformation. Indeed, one could imagine that mAb 20.1-binding “freezes” BTN3-ED in a conformation (93), which is distinct from the PAg-induced one (93, 95). Considering inherent qualities of TCR clonotypes as the basis for their differential capacity in recognizing BTN3A1-ED-PAg complex or BTN3-mAb complex, we propose or speculate that some Vγ9Vδ2 TCR, e.g., TCR B2G9 preferentially bind to a complex of PAg bound to the BTN3A1-ED, whereas others would preferentially bind to the conformationally changed BTN3A1 whose ED does not need to be in complex with the PAg. Consistent with this model would be that the area covered by the mAb 20.1 is rather near to the hypothetical PAg-binding site discussed in the next paragraph. Consequently for some TCR mAb 20.1 would compete with binding of the Vγ9Vδ2 TCR to a BTN3A1-PAg complex while for others mAb 20.1 would still be stimulatory.

De Libero and coworkers (95) provide also a wealth of data in favor of a direct binding of PAg to BTN3A1-ED and of binding of BTN3A1-PAg complexes to the Vγ9Vδ2 TCR: (i) IPP and HMBPP induce a substantial IFNγ secretion by the murine reporter cells cultured in BTN3A1-V domain coated culture plates. (ii) Mass spectrometry data of BTN3A1-V incubated with IPP is consistent with a BTN3A1-IPP complex of 1:1 stoichiometry. (iii) Plasmon resonance analysis of PAg binding to BTN3A1-V domain allowed calculation of *K*_d_ values. These are considerably lower than that of MHC-peptide complexes: 66.9 × 10^−6^ M for binding of BTN3A1-V to IPP and 3.06 × 10^−6^ M for binding of BTN3A1-V to HMBPP. (iv) Crystal structures of complexes generated from BTN3A1-V and IPP or HMBPP, respectively, identify a shallow Ag-binding groove. The amino acids proposed to interact with HMBPP or IPP respectively are marked in Figure 1A. (v) Plasmon resonance analysis revealed a low affinity binding of recombinant Vγ9Vδ2 TCR multimers (dextramers) (TCR G2B9) to immobilized eukaryotic recombinant BTN3A1-ED (vi) Surface enhanced Raman scattering demonstrates binding of monomeric TCR to BTN3A1 with a *K*_d_ of 34 × 10^−5^ M in the presence and 93 × 10^−5^ M in the absence of IPP. All these data can be interpreted as evidence that BTN3A1 serves an antigen-presenting molecule (95). A simplified version of this hypothesis is depicted in Figure 2B.

The results and conclusions (95) on BTN3-binding to PAg and Vγ9Vδ2 TCR summarized in the previous paragraph are heavily disputed by proponents of the “allosteric model” (94). In their recent publication, the groups of Adams and Scotet/Bonneville, respectively, give detailed account on failed attempts to detect PAg-binding to the BTN3-ED and of Vγ9Vδ2 TCR (TCR-G115) dextramers to immobilized BTN3-ED. Furthermore, their paper opposes the interpretation of the BTN3-V domain crystallized in presence of PAg as BTN3-PAg complex (94). Based on their analyses of the B30.2 domain and their own negative data on PAg- and TCR-binding, BTN3A1 is refuted as an “*antigen-presenting molecule*.” Instead, they propose in the discussion section: “a model where PAg binding to the BTN3A1 ID results in recruitment of additional primate-specific factors and/or rearrangement of the BTN3A1 extracellular domain that generates a stimulatory signal directly detected by the Vγ9Vδ2 TCR. This is similar to “Model 2” proposed by Morita and colleagues” (94). The paper of the Morita group (80) mentioned here describes their failed attempts to show binding to BTN3-ED by the use of photo-activatable HMBPP and develops models to explain lacking PAg-binding to BTN3-ED despite its crucial role for the activation of Vγ9Vδ2 T cells.

Some of the data and interpretations reported by the proponents of either model can be reconciled and others not. For example, both agree on the importance of the ID for BTN3A1 mediated PAg activation, and in principle the data on PAg-binding to the BTN3-ID could be adapted in a model with BTN3A1 as an antigen-presenting molecule in following ways: (1) PAg-binding to the BTN3A1-ID may result in intracellular trafficking, e.g., recruitment of a molecule which enables loading of PAg to the BTN3A1-ED and/or involved in proper cell surface distribution of BTN3A1 with the PAg loaded BTN3A1-ED. (2) Another possibility could be that PAg-binding to the BTN3A1-ID induces a conformational change facilitating formation of the BTN-ED-PAg complex or stabilization of such a complex. Concerning the dissent on crystallographic and binding data it would be most desirable – and important for the research community – if controversies on the validity of experimental procedures and their interpretation were solved, e.g., by collaborative attempts of both parties to reproduce data with exchanged reagents, e.g., the different BTN3 preparations and the different TCR clonotypes (TCR-G115 vs. TCR-G2B9). In addition, completely different experimental approaches such as the identification of cofactors controlling PAg-mediated activation by genetic methods might lead to new insights and perspectives and help to solve this controversy.

## Evidence for Chr 6 Encoded Gene(s), Which in Addition to BTN3A1 are Mandatory for PAg-Mediated Activation

A first hint that BTN3A1 alone is not the only molecule expressed in primates required for PAg mediated activation comes from experiments where BTN3A1-transduced murine cells pulsed with zoledronate failed to activate human Vγ9Vδ2 T cells (94). Nevertheless, these negative results need to be met with caution since in this xenogeneic setting co-stimulatory receptors and adhesion molecules required for Vγ9Vδ2 TCR-mediated activation might miss their partners on the murine cell and activation may be incomplete.

To solve this problem, murine reporter cells provide a valuable tool. We tested mouse-human hybridomas generated in our laboratory for their capacity to activate reporter cells (TCR-MOP transductants) (97) similarly, as it was done in (95). Consistent with the localization of *BTN3A1* on chromosome 6 (Chr. 6) only hybridoma carrying the human Chr. 6 were able to induce PAg-dependent Vγ9Vδ2 TCR responses. To test whether *BTN3A1* alone or *BTN3A1* and other genes on Chr. 6 allow the PAg-mediated stimulation and whether the same accounts for mAb 20.1-induced activation, different types of Chinese hamster ovary (CHO) cells were tested. We compared CHO cells and CHO cells containing human Chr. 6 and the *BTN3A1* transductants of either cell type. In a nutshell, Chr. 6 was found to be sufficient and mandatory to induce activation in the presence of HMBPP and zoledronate while BTN3A1 alone allowed mAb 20.1-induced activation even in the absence of Chr. 6 (97).

These data as well inhibition studies with BTN3-specific antibodies are in full agreement with the pivotal role of BTN3A1 in PAg-mediated activation but imply also the existence of one or more human gene(s) controlling PAg-action, which are missing in rodent cells. Such gene(s) could be involved in PAg-loading onto BTN3A1 analogous to the TAP being mandatory for MHC-class I mediated peptide-presentation. Other possibilities would be, e.g., control of BTN3A1 related signaling pathways steering cellular distribution of BTN3A1 or a factor X directly associated with BTN3A1. In any case identification of these molecules in primates will be essential for the design of rodent models with functional Vγ9Vδ2 T cells, which can be expected to provide fresh insights of Vγ9Vδ2 T cell physiology and facilitate preclinical research on Vγ9Vδ2 T cells and Vγ9Vδ2 T cell-activating drugs (97). The search for such molecule(s) could be facilitated by the murine reporter cells (95, 97), which allow functional screens for the missing genes, e.g., of rodent-human radiation hybrids containing fragments of human Chr. 6 or human expression libraries.

An important implication of the finding that mAb 20.1 permits Vγ9Vδ2 TCR-mediated activation by BTN3A1 expressing rodent cells is that species (human) specific gene(s) can be dispensable for PAg-independent Vγ9Vδ2 TCR-mediated activation. We suggest that mAb 20.1 induces a conformational change of BTN3A1, which allows the Vγ9Vδ2 TCR to directly interact with BTN3A1 or a highly conserved molecule, which upon mAb 20.1 incubation interacts with BTN3A1 and is then recognized by the TCR.

We tested also CD69 up-regulation of human Vγ9Vδ2 T cells with PBMC co-cultured with different CHO cell variants pulsed with zoledronate. Only co-cultures with Chr. 6 consomic CHO cells induced a Vγ9Vδ2 T cell specific activation. Interestingly, we failed to detect CD107a induction suggesting that in this setting Vγ9Vδ2 TCR-mediated activation is incomplete (97). This is of interest since Sandstrom et al. (94) who tested another xenogeneic setting namely co-culture of primary Vγ9Vδ2 T cells with zoledronate or mAb 20.1 pulsed BTN3A1-transduced murine cells used CD107a expression as read out and might have missed a partial activation. Not excluded can be that BTN3A1-transduced CHO cells, which are of hamster origin might express species- or cells-specific factors that are missing in murine BTN3A1 transductants (94) and allowed activation of our reporter cells (TCR-MOP transductant).

## Vγ9Vδ2 TCR: The Neglected Interaction with Protein Antigens

The identification of PAg as activator for the vast majority of Vγ9Vδ2 T cells does not exclude other physiological modes of TCR-triggered Vγ9Vδ2 T cell activation. In a series of studies He and colleagues investigated tumor infiltrating γδ T cells, i.e., especially malignancies of ovarial origin, and identified specific ligands whose recognition depended largely on the CDR3δ and CDR3δ flanking regions of the TCR (98–103). This was demonstrated with help of TCR transductants but also with Ig constructs with implanted CDR3δ, which bind to tumors *in vitro* and trigger tumor elimination in xenografted mice (103). Among the proposed antigens were MutS homolog 2, hsp 60 (99), and the NKG2D ligand ULBP-4 (100). In one case tumor-specific and PAg-mediated activation were compared and mutagenesis of amino acid 97 in the CDR3δ abolished PAg but not an anti-tumor response (102). This CDR3δ-controlled recognition is reminiscent of that of T22 molecules by murine γδ TCR (12, 104) and could also be important for other human γδ T cells. In any case, apart from its therapeutic potential the possibility of PAg-independent tumor recognition by Vγ9Vδ2 T cells demonstrates a function of Vγ9Vδ2 T cells beyond that of a sensor of metabolic aberrations or of microbial metabolites (69). Indeed, it is rather likely that such (oligo)clonal responses might have been missed in the analysis of PAg-responding cells given the presumed low frequency of such protein antigen-specific Vγ9Vδ2 TCR in comparison to PAg-reactive cells. Furthermore, these oligoclonal PAg-independent responses might contribute to the changes in the Vγ9Vδ2 T cell repertoire seen during infections with HIV or with *Mycobacterium tuberculosis* (49, 105).

## Vγ9Vδ2 TCR and BTN3 in Primates

Monkeys including hominids possess Vγ9Vδ2 TCR and functional *BTN3A1* genes (80, 93, 106, 107). PAg reactivity of Vγ9Vδ2 T cells has been directly demonstrated for simian species (simiiformes), which include the new world monkey Nancy Ma’s night monkey (*Aotus nancymaae*) (108) and the common marmorset (*Callithrix jacchus*) (109) and the old world monkeys rhesus macaque (*Macaca mulatta*) (110) and cynomologous macaque (*Macaca fascicularis*) (111), which bear a hydrophobic amino acid at position 97 of delta chain and Vγ9JP rearrangements. Human and most primate species have a K(Lysine)KIK motif in JP, which is sometimes changed to R(Arginine)KIK without apparent consequences for PAg reactivity (107, 110).

An update in September 2014 of our previous searches (107) identified translatable Vγ9, Vδ2, and BTN3A1 sequences in lower primates for the representative of non-simiiforme haplorrhini, the philippine tarsier (*Carlito syrichta*). The same was found for aye aye (*Daubentonia madagascariensis*) and the gray mouse lemur (*Microcebus murinus*) representing the two lemur clades of the strepsirrhini suborder. The BTN3A1 sequences were largely conserved in the proposed PAg-binding motif of the BTN3-ED and in the BTN3-ID. The BTN3-ID of aye aye and tarsier showed the H351R substitution, which abrogates PAg-binding of BTN3A3 (Figure 1). Therefore, it would be interesting to learn whether this also leads to loss of PAg-binding or is compensated by other sequence changes. If PAg-binding of the BTN3-ID was indeed lost and if PAg involvement is needed to maintain the typical Vγ9Vδ2 TCR repertoire then one would expect consequential effects on this repertoire, e.g., less or no Vγ9JP rearrangements.

## Vγ9, Vδ2, and *BTN3* Co-Emerged and may have Co-Evolved in Placental Mammals

Studies on rodent genomes and on TCR expressed in farm animal species (cow, pig, horse) provided no evidence for Vγ9 or Vδ2 homologs, and it was generally assumed that Vγ9Vδ2 T cells might be restricted to (higher) primates. Taking advantage of increasing number of public data genome data bases, especially the 29 mammals project, which covered all mammalian orders (112), we blasted against genomes of Eutherian mammalian species in search for genes homologous to human Vγ9, Vδ2, and *BTN3-ED*. We asked for at least 80% sequence coverage and 70% nucleotide identity to human genes in order to identify new species as potential Vγ9Vδ2 T cell carriers. Homology was confirmed by reverse blasting and different types of phylogenetic trees were generated. Most of these results have been published recently (107) but we take the opportunity of this paper to present changes resulting from recent updates in the database and from inclusion of the BTN3-ID in our analysis (Figure 1).

Truly surprising results were obtained from the analysis of non-primate species. The timing of the origin of placental mammals is subject of a protracted debate and has been estimated to occur between 165 and 65 million years ago (113–115). Nevertheless, there is consensus that Xenathra – represented in the databases by the nine-banded armadillo *(Dasypus novemcinctus)* and the two fingered sloth (*Choloepus hoffmanni*) belong to a clade distinct from Boreoeutheria, which represents the other placental mammals. Thus, detection of a gene in a species of Xenathra and Boreoeutheria proves its presence in a common placental predecessor. Exactly, this is seen for Vγ9, Vδ2, and *BTN3-ED* since they are found in sloth and armadillo. While *TCR-V* genes were rather conserved (107), Figure 1 shows that *BTN3-ED* of sloth lacks a major part of the V domain while that of armadillo lacks parts of the C domain. A *BTN3-ID* like domain could not be identified for sloth and the B30.2 domain of armadillo was identified as a homolog of *BTN2-ID*. With the current knowledge on BTN3-structure-function relationship and PAg-mediated Vγ9Vδ2 T cell activation, it seems unlikely that these molecules could function as proposed for human BTN3. Consequently, we would not expect maintenance of typical Vγ9Vδ2 T cells (e.g., dominance of the characteristic Vγ9JP rearrangements) but if such cells were found, then they should be tested for PAg-reactivity or -binding to other ligands. Testing is especially relevant for armadillo, since it is a natural host *Mycobacterium leprae*, an important human pathogen and potential target for Vγ9Vδ2 T cells (116).

Many of the tested genomes had lost all three genes. This was true for lagomorphs and most rodents and explains why PAg-reactive cells have never been observed in the classical small animal models (rat, mouse, guinea pig, and rabbit). A notable exception could be the 13-lined squirrel (*Spermophilus tridecemlineatus*), which conserved a translatable Vγ9 and a presumably functional BTN3 (Figure 1) but lacks a Vδ2. Therefore, it will be of special interest to learn whether this Vδ2 is truly missing or whether the searched database is incomplete. Species of other orders such as those representing Bovidae (cow, sheep, goat, tibetan antelope) kept all three genes but at least one of them was either not translatable (e.g., *BTN3-ID* of tibetan antelope) or otherwise non-functional as in the case of the horse (Perissodactyla) whose Vγ9 and Vδ2 genes lacked one of the cysteine required for Ig domain disulfide bond (107).

It is very striking that all species (11 species representing 9 families representing 3 of the 4 mammalian superorders) with translatable Vγ9 and Vδ2 TCR gene possess translatable *BTN3-ED*. The inverse correlation is not as strict, since BTN3A1 of horse (Equidae), white rhinoceros (Rhinocerotidae), several bats (Vespertilionidae), and the thirteen-lined squirrel (Sciuridae) are translatable, although the species lack either functional Vγ9 and/or Vδ2 genes. However, inspection of BTN3-ID (Figure 1) of horse, rhinoceros and bats shows considerable differences in the proposed PAg-binding site suggesting loss of PAg-related function. These species show non-conservative substitutions in the proposed extracellular and intracellular PAg-binding sites while the other species with a complete and translatable BTN3-ED showed no or only conservative changes (K to R and vice versa). Altogether, the data suggest an interdependence in conservation of function between Vγ9, Vδ2, and *BTN3* genes and indicate molecular co-evolution of Vγ9Vδ2 TCR and BTN3 (117).

### Alpaca (*Vicugna pacos*) as model to study Vγ9Vδ2 T cells in a non-primate species

Of the nine species originally found to possess translatable Vγ9, Vδ2, and *BTN3-ED* genes (107), alpaca was the only one accessible for further analysis. Vγ9-Cγ PCR products from PBMC cDNA were cloned and although different Jγ segments were identified, 90% of the clones showed a JP rearrangement. The primate KKIK or RKIK motif is largely conserved in the three alpaca JP, which have a KTIK or RTIK motif. In contrast to human Vγ9Vδ2 T cells, the Vδ2 gene rearranged (25 out of 25 clones) always with a single Jδ gene, which is highly homologous to human Jδ4 (TRDJ*04.) The amino acids L, I, V, and G at position 97, which are typical for PAg reactive human Vγ9Vδ2 TCR were found in 8 out of 17 clones bearing alpaca Vδ2 chains. Similar to human, alpaca Vδ2 chains also show high diversity in CDR3 lengths ranging from 11 to 18 amino acids (38, 107), while CDR3γ length is rather restricted (107). Transduction of 58α-β-cells (BW58) with full length alpaca Vγ9 and Vδ2 TCR chain genes led to their cell surface expression as assessed by surface staining for murine CD3 and detection of IL-2 upon culture with immobilized anti-CD3 antibodies. Due to lack of appropriate reagents, the formal proof of cells concomitantly expressing Vγ9 and Vδ2 TCR chains is still missing.

Nevertheless, at least with respect to *BTN3A1* and the TCR genes, there is currently no obvious reason to assume that PAg could not activate alpaca Vγ9Vδ2 T cells. Indeed, the BTN3A1 has been cloned and expressed and showed complete identity with human BTN3 in the published intracellular- and extracellular PAg-binding sites (118). The functionality with respect to PAg-responses will be tested with γδ TCR transductants and camelid stimulator cells but also with alpaca PBMC.

If PAg-reactivity can be confirmed, alpaca will provide an outgroup allowing identification of common denominators of PAg-reactivity of Vγ9Vδ2 T cells, and it will allow analysis of conservation of the molecular mechanisms of PAg-dependent stimulation or presentation, respectively. If PAg-reactivity is missing, then interesting questions arise. Are there alternative modes of Vγ9Vδ2 T cell activation and are they also BTN3A1-dependent? If there is a BTN3-dependent Vγ9Vδ2 T cell activation, new questions arise such as, did other molecules take over the role of PAg and can introduction of human genes (favorably encoded on chromosome 6) generate a BTN3A1-dependent PAg-response similar to that seen in humans?

## Identifying Candidate Species for Dendritic Epidermal T Cells by Investigating γδ TCR Genes and *Skint1* Homolog in Mammals

**Figure 3:**
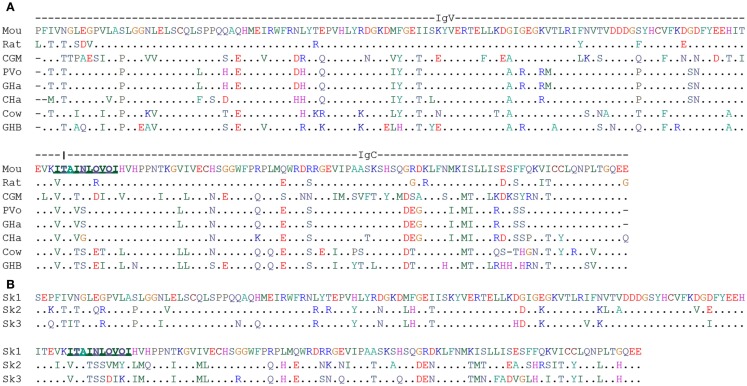
**Alignment of human BTN3A1 (V-C) and B30.2 domain**. **(A)** ClustalW2 amino acid alignment of V-C domains of mouse Skint1 extracellular domain with respective homologous sequence identified from WGS database at NCBI. Underlined bold 10 amino acids stretch is predicted to confer functionality for mouse Skint1 (122). **(B)** ClustalW2 amino acid alignment of V-C domains of mouse Skint1 extracellular domain with its paralogs. Underlined bold 10 amino acids stretch is predicted to confer functionality for mouse Skint1 (122). Species were abbreviated as Mou, Mouse; CGM, Cape golden mole; PVo, Prairie vole; GHa, Golden hamster; CHa, Chinese hamster; GHB, Greater horseshoe bat.

At the end of this review, we want to discuss a possible co-evolution of γδ TCR genes and their relation to another member of the butyrophilin family. Dendritic epidermal T cells (DETC) present an extreme case of a highly specialized γδ T cell population, which so far has only been found in mice and rats (28, 119, 120). As the name indicates these T cells have dendritic shape and reside in the epidermis. They appear as the earliest T cells during development in the fetal thymus and subsequently migrate to skin. In the skin, they fulfill TCR-dependent and TCR-independent functions in body barrier surveillance including control of tumor development, skin repair, and allergy control. A hallmark of these cells is expression of a single TCR, which with unique rearrangements containing Vγ3 and Vδ4 (IMGT-nomenclature; other common nomenclatures are Vγ3Vδ1 and Vγ5Vδ1). Canonical DETC can be replaced by cells with a polyclonal TCR repertoire but these do not fulfill all of their functions (15, 25, 30). Mandatory for the development of canonical DETC is the molecule Skint1, which is a member of the butyrophilin family. It consists of a V-C domains containing extracellular domain and a three times transmembrane-spanning domain (31, 121, 122). Its role in DETC development was discovered by analysis of FVB/N mice from Taconics laboratories. These mice showed changes in the DETC TCR repertoire, which were correlated with a skin phenotype (spontaneous ear inflammation and exaggerated irritant contact dermatitis response to tetradecanoyl-phorbol acetate). The genetic basis of this phenotype is a termination mutation in codon 324 of Skint1 immediately upstream of the third transmembrane domain of the molecule (31, 121). In a recent study on structure-function relationship of Skint1, it was found that a tightly regulated cell surface expression on medullary thymic epithelial cells was mandatory for efficient DETC development. Furthermore, mutagenesis and domain exchange proved that each Skint1 domain is non-redundant, including a unique decamer specifying V-domain processing (122) (marked in Figure 3).

Prompted by the striking concomitant conservation of *TCR-V* genes and *BTN3A1* and the success with identification of alpaca as a candidate for a Vγ9Vδ2 T cell positive species, we decided to search for homologs of DETC *TCR-V* genes Vγ3 and Vδ4 and of *Skint1* as a gene controlling DETC development using the same methods as for *BTN3* and Vγ9Vγ2 TCR genes (107). Homologs for at least one of the three genes were identified in 69 species, all of them were Eutheria. No hits were found for Xenathra but the cape golden mole was identified as an afrotherian species being “triple positive.” In case of the *TCR-V* genes, the assignment was always clear. In case of *Skint1*, forward blasting of the V-C domains let to identification of genes as *Skint1* homolog, which in reverse blast turned out to be *Skint2* or *Skint3* homologs. We marked this in our table (Table S1 in Supplementary Material) but the limited knowledge about Skint structure and mode of action does not allow to make any predictions to which extent the Skint homologs in these species are redundant – which at least in mouse they are not (122). Also statements on translatability have to be taken with some caution since transmembrane encoding exons were not included in the search.

Nevertheless, the retrieved data (Table S1 in Supplementary Material and Figure 3) could help to identify non-murine species as candidates for a search for rearranged DETC TCR genes and functional Skint: prime candidates are rodent species, which do not belong to the family of murideae but are still phylogenetically not too distant. These would be the two hamster species. Also interesting but unfortunately not yet sequenced at genomic level is the cotton rat (*Sigmodon hispidus*) and therefore not to be found in the Table S1 in Supplementary Material and Figure 3. This “new world mouse” species is a well-established animal model for a number of infectious diseases (123). Triple positive members were also found in other mammalian superorders than the rodent-containing Euarchontoglires. These are Afrotheria with the golden cape mole (*Chrysochloris asiatica*) and Laurasiatheria species with the greater horseshoe bat (*Rhinolopus ferrumequinum*) and the cow (*Bos tauris*). Nearly, all bats (Chiroptera) carry translatable Vγ3, Vδ4, and either Skint1 or Skint2. Finally, the cow was also triple positive and was the only Artiodactyla species with a translatable Skint. Given the limits of our data-base search, however, at first it needs to be tested whether the latter is still functional. If expression of a Skint1 can be confirmed, it will be of interest to test whether a cow DETC population might be hidden among other populations such as the circulating polyclonal dermal γδ T cells or the small epidermal γδ T cell population (124).

Nevertheless, despite all possible pitfalls in database analysis, we are confident that comparative analysis of TCR genes and genes of putative TCR ligands or of molecules controlling development and function of non-conventional T cells will allow to identify genetic or functional homologs to human non-conventional T cells. The identification of such homologous populations in phylogenetic distant species or species with different life style could help to identify common themes on preservation and flexibility of genes and of functions of such cells. Going back to human beings (or mice) and seeing old acquaintances in a new light may help for a better understanding of the human system and identification of targets of genetic and immune-intervention.

## Conflict of Interest Statement

The authors declare that the research was conducted in the absence of any commercial or financial relationships that could be construed as a potential conflict of interest.

## Supplementary Material

The Supplementary Material for this article can be found online at http://www.frontiersin.org/Journal/10.3389/fimmu.2014.00648/abstract

## Abbreviations

aa, amino acids; BTN, butyrophilin; BTN3A1-ED, butyrophilin-3 A1 extracellular domain; BTN3A1-ID, butyrophilin-3 A1 intracellular domain; Chr. 6, chromosome 6; DETC, dendritic epidermal T cells; DOXP, 1-deoxy-d-xylulose 5-phosphate pathway; FPPS, farnesyl diphosphate synthase; HMBPP, (E)-4-hydroxy-3-methyl-but-2-enyl pyrophosphate; IPP, isopentenyl pyrophosphate; mAb, monoclononal antibody; MEP, 2-C-methyl-d-erythritol 4-phosphate pathway; PAg, phosphoantigen; VLR, variable leukocyte receptors.
