# Supplementary material for: The Vγ9Vδ2 T Cell Antigen Receptor and Butyrophilin-3 A1: Models of Interaction, the Possibility of Co-Evolution, and the Case of Dendritic Epidermal T Cells
Source: Front Immunol. 2014 Dec 19;5:648. doi: 10.3389/fimmu.2014.00648 (PMC4271611; doi:10.3389/fimmu.2014.00648)
Supplement: Supplementary file 1 [file Table_1.DOC]

***Supplementary table***

**The Vγ9Vδ2 T cell antigen receptor and butyrophilin-3 A1: models of interaction, the possibility of co-evolution, and the case of dendritic epidermal T cells**

**Mohindar M. Karunakaran^1^ and Thomas Herrmann^1*^**

^1^Institute for Virology and Immunobiology, Dept of Medicine, University of Würzburg, Germany

***Correspondence:** Prof. Dr. Thomas Herrmann, Institut für Virologie und Immunbiologie,

Versbacher Strasse 7, 97078 Würzurg, Germany.

E-mail: herrmann-t@vim.uni-wuerzburg.de

1. **Supplementary tables**

**Supplementary Table 1: Distribution of Vγ3, Vδ4 and *Skint1 (V-C)* genes among placental mammals.** The table represents the distribution of Vγ3, Vδ4 and *Skint1 (V-C)* * genes across the mammals belonging to each superorders . Mouse genes were taken as query and blasted against the NCBI whole genome database (Whole-genome Shotgun database). The homologous and paralagous nucleotide sequences from different species were indicated by the percentage of identity and sequence coverage length to that of query sequences. Asterisk represents the species which carries translatable Vγ3, Vδ4 and *Skint1 (V-C)* genes. Underlined numbers indicate the non-translatable gene sequences. Accession numbers of the sequences can be provided upon request. *V-C indicates those exons of the skint genes encoding for the IgV and IgC like domains of skint. Sk1, Sk2 and Sk3 are skint paralogues.

| Super Order | Clade | Order | Family | Species | Vγ3 (Identity/ Cover %) | Vδ4  (Identity/ Cover %) | *Skint1(V-C)*  (Identity/ Cover %) |
| --- | --- | --- | --- | --- | --- | --- | --- |
| Afro-theria | Afro-insecti-philia | Afro-soricida | Tenrecidae | *Echinops* *telfari*  (The Lesser hedgehog tenrec) | - | - | - |
|  |  |  | Chryso-cloridae | *Chrysochloris* *asiatica* (Cape golden mole) * | 80/100 | 77/98 | 72/99 (Sk1) |
|  | Paen-ungulata | Hyra-coidea | Procaviidae | *Procavia* *capensis* (Rock hyrax) | 76/100 | 71/94 | - |
|  |  | Probo-scidea | Elephan-tidae | *Loxodonta* *Africana*  (African elephant) | 79/100 | - | - |
|  |  | Sirinia | Triche-chidae | *Trichechus* *manatus* *latirostris* (The Florida manatee ) | - | 75/98 | - |
| Xenar-thra |  | Cingulata | Dasypodi-dae | *Dasypus* *novemcinctus* (Nine-banded armadillo) | - | 73/96 | - |
|  |  | Pilosa | Megalony-chidae | *Choleopus* *hoffmanni* (Sloth) | - | 73/98 | - |
| Euar-chonto-glires | Euarchonto | Scanden-tia | Tupaiidae | *Tupaia* *belangeri* (The northern treeshrew) | 78/97 | 78/97 | 76/99 (Sk2) |
|  |  | Primata | Dauben-toniidae | *Daubentonia* *madagascariensis*  (Aye-aye) | 82/94 | 76/97 | - |
|  |  |  | Tarsiidae | *Carlito* *syrichta* (Philippine tarsier) | 82/100 | 75/96 | 76/98 (Sk2) |
|  |  |  | Cebidae | *Saimiri* *boliviensis* *boliviensis* (Black capped squirrel monkey) | 76/94 | 73/98 | - |
|  |  |  | Cercopithe-cidae | *Macaca* *Mulatta* (Rhesus Monkey) | 82/100 | 73/98 | 78/99 (Sk2) |
|  |  |  |  | *Macaca* *fascicularis* (Crab eating Macaque) | 82/100 | 74/98 | 78/99 (Sk2) |
|  |  |  |  | *Chlorocebus* *sabaeus* (Green monkey) | 83/95 | 73/98 | 77/99 (Sk2) |
|  |  |  |  | *Papio* *anubis* (Olive baboon) | 83/95 | 73/98 | 78/99 (Sk2) |
|  |  |  | Hyloba-tidae | *Nomascus* *leucogenys* (Gibbon) | - | 74/96 | 77/100 (Sk2) |
|  |  |  | Hominidae | *Pongo* *abelii* (Sumatran Oranguttan) | - | 76/98 | 78/99 (Sk2) |
|  |  |  |  | *Gorilla* *gorilla* (Gorilla) | - | - | 78/99 (Sk2) |
|  |  |  |  | *Pan* *paniscus* (Bonobo) | 83/95 | 74/98 | 78/99 (Sk2) |
|  |  |  |  | *Pan* *troglodytes* (Chimpanzee) | 83/95 | 74/98 | 56/80 (IgV) |
|  |  |  |  | *Homo* *sapiens* (Human) | - | - | 77/99 (Sk2) |
|  | Glires | Lago-morpha | Ocho-tonidae | *Ochotona* *princeps* (The America Pika) | 79/100 | 74/84 | 74/98 (Sk2) |
|  |  |  | Leporidae | *Oryctolagus* *cuniculus* (The European Rabbit) | 77/100 | 74/98 | - |
|  |  | Rodentia | Bathyer-gidae | *Heterocephalus* *glaber* (Naked mole rat) | 81/100 | 76/95 | - |
|  |  |  | Caviidae | *Cavia* *porcellus* (Guinea pig) | - | - | - |
|  |  |  | Cricetidae | *Peromyscus* *maniculatus* *bairdii* (Deer mouse) | 82/98 | 82/98 | 82/98 (Sk3) |
|  |  |  |  | *Cricetulus* *griseus* (Chinese hamster) * | 90/100 | 82/98 | 83/99 (Sk1) |
|  |  |  |  | *Mesocricetus* *auratus* (Golden Hamster) * | 83/94 | 81/98 | 80/95 (Sk1) |
|  |  |  |  | *Microtus* *ochrogaster* (Prairie vole) * | 88/100 | 83/98 | 83/99 (Sk1) |
|  |  |  | Muridae | *Mus* *musculus* (Mouse) * | 100/100 | 100/100 | 100/100 |
|  |  |  |  | *Rattus* *norvegicus* (Rat) * | 94/100 | 90/100 | 91/98 (Sk1) |
|  |  |  | Sciuridae | *Spermophilis* *tridecemlineatus* (Thirteen lined Squirrel) | 80/100 | 77/98 | 76/98 (Sk3) |
| Laur-asia-theria | Euli-potyphla | Erinaceo-morpha | Erinaceidae | *Erinaceus* *europaeus* (European hedgehog) | 78/95 | 69/100 | - |
|  |  | Sorico-morpha | Soricidae | *Sorex* *araneus* (Common Shrew) | 77/100 | 74/96 | 74/89 (Sk2) |
|  | Scroti-fera | Artio-dactyla | Camelidae | *Vicugna* *pacos* (Alpaca) | - | 74/98 | - |
|  |  |  |  | *Camelus* *ferus* (Wild Bactrian Camel) | 75/100 | 74/98 | - |
|  |  |  | Bovidae | *Bos* *taurus* (Cow) * | 82/95 | 74/97 | 77/99 (Sk1) |
|  |  |  |  | *Bubalus bubalis* (Water buffalo) | 82/95 | 75/98 | 76/98 (Sk1) |
|  |  |  |  | *Ovis* *aries* (Sheep) | 82/100 | 75/100 | 78/88 (Sk2) |
|  |  |  |  | *Capra* *hircus* (Yunnan black goat) | 80/100 | 72/98 | 77/98 (Sk2) |
|  |  |  |  | *Pantholops* *hodgsonii* (Tibetan antelope) | 83/95 | 73/98 | 78/88 (Sk2) |
|  |  |  | Suidae | *Sus* *scrofa* (Wild Boar) | 79/100 | 75/94 | 71/99 (Sk1) |
|  |  | Cetacea | Delphini-dae | *Tursiops* *truncates*  (Bottle nose Dolphin) | - | 77/96 | 76/99 (Sk1) |
|  |  |  |  | *Orcinus* *orca* (Killer whales) | - | - | 75/99 (Sk1) |
|  |  | Chiroptera | Ptero-podidae | *Pteropus* *vampyrus* (Flying Fox) | 82/95 | 77/97 | 78/55 (Sk3) |
|  |  |  |  | *Pteropus* *alecto* (Black Flying fox) | 82/100 | 76/97 | 76/98 (Sk2) |
|  |  |  |  | *Eidolon* *helvum* (Straw coloured bat) | 83/95 | 76/97 | 76/98 (Sk2) |
|  |  |  | Mormoopi-dae | *Pteronotus* *parnellii* (Parnell’s mustached bat) | 82/95 | 76/98 | 76/99 (Sk2) |
|  |  |  | Megader-matidae | *Megaderma* *lyra* (Greater false Vampire bat) | 82/100 | 73/98 | 75/95 (Sk2) |
|  |  |  | Rhinolophi-dae | *Rhinolophus* *ferrumequinum* (Greater horseshoe bat) * | 80/100 | 77/98 | 79/97 (Sk1) |
|  |  |  | Vespertilio-nidae | *Myotis* *lucifugus* (Fruit bat) | 80/100 | 78/97 | 76/99 (Sk2) |
|  |  |  |  | *Myotis* *brandtii* (Brandt’s bat) | 80/100 | 78/98 | 77/99 (Sk2) |
|  |  |  |  | *Eptesicus* *fuscus* (Big brown bat) | 80/100 | 76/98 | 77/99 (Sk2) |
|  |  | Perisso-dactyla | Equidae | *Equus* *caballus* (Horse) | - | 77/96 | - |
|  |  |  | Rhinocero-tidae | *Ceratotherium* *simum* (White Rhinoceros) | 72/100 | 73/98 | - |
|  |  | Carnivora | Felidae | *Felis* *catus* (Cat) | 70/100 | 72/98 | - |
|  |  |  | Canidae | *Canis* *familaiaris* (Dog) | 70/100 | 73/93 | - |

1. Springer MS, Stanhope MJ, Madsen O, de Jong WW. Molecules consolidate the placental mammal tree. Trends in ecology & evolution. 2004;19(8):430-8.
